# Mapping of MN1 Sequences Necessary for Myeloid Transformation

**DOI:** 10.1371/journal.pone.0061706

**Published:** 2013-04-23

**Authors:** Ayten Kandilci, Jacqueline Surtel, Laura Janke, Geoffrey Neale, Sabrina Terranova, Gerard C. Grosveld

**Affiliations:** 1 Department of Genetics, St Jude Children’s Research Hospital, Memphis, Tennessee, United States of America; 2 Veterinary Pathology Core, St Jude Children’s Research Hospital, Memphis, Tennessee, United States of America; 3 Hartwell Center for Bioinformatics and Biotechnology, St Jude Children’s Research Hospital, Memphis, Tennessee, United States of America; Cincinnati Children’s Hospital Medical Center, United States of America

## Abstract

The *MN1* oncogene is deregulated in human acute myeloid leukemia and its overexpression induces proliferation and represses myeloid differentiation of primitive human and mouse hematopoietic cells, leading to myeloid leukemia in mouse models. To delineate the sequences within MN1 necessary for MN1-induced leukemia, we tested the transforming capacity of in-frame deletion mutants, using retroviral transduction of mouse bone marrow. We found that integrity of the regions between amino acids 12 to 458 and 1119 to 1273 are required for MN1’s *in vivo* transforming activity, generating myeloid leukemia with some mutants also producing T-cell lympho-leukemia and megakaryocytic leukemia. Although both full length MN1 and a mutant that lacks the residues between 12–228 (Δ12–228 mutant) repressed myeloid differentiation and increased myeloproliferative activity *in vitro*, the mutant lost its transforming activity *in vivo*. Both MN1 and Δ12–228 increased the frequency of common myeloid progentiors (CMP) *in vitro* and microarray comparisons of purified MN1-CMP and Δ12–228-CMP cells showed many differentially expressed genes including Hoxa9, Meis1, Myb, Runx2, Cebpa, Cebpb and Cebpd. This collection of immediate MN1-responsive candidate genes distinguishes the leukemic activity from the in vitro myeloproliferative capacity of this oncoprotein.

## Introduction

Acute myeloid leukemia (AML) in adults is a leading cause of leukemia-related deaths, and is characterized by uncontrolled proliferation and impaired differentiation of hematopoietic cells that results in accumulated myeloid blasts in the bone marrow and periphery. [1] Tight control of the balance between proliferation and differentiation is essential for the maintenance of normal hematopoeisis. In various AML subtypes, deregulation of different or sometimes overlapping genes disrupts this balance and causes AML. [1], [2] These genes mostly control the survival/proliferation/differentiation programs of the hematopoietic stem/progenitor cells (HSPC). [1], [2].

*MN1* (Meningioma 1) is located on human chromosome 22 and encodes a 1319 amino acid (aa) long protein, which is unique as it does not show homology to any known proteins. [3] MN1 is involved in AML either as a partner of the t(12;22)(p12;q12), creating an MN1-TEL fusion protein, [4] or as an overexpressed gene.[5]–[7] About half of patients with AML carry leukemic cells with a normal karyotype [8] in which elevated *MN1* expression correlates with poor prognosis. [9] In addition, increased expression of MN1 cooperates with CBFβ-SMMHC [7], NUP98-HOXD13 [10] and MLL-ENL [11] fusion proteins to induce leukemia, suggesting that deregulation of *MN1* expression contributes to leukemogenesis. Indeed, others and we have shown that ectopic expression of MN1 in mouse HSPC (Hematopoietic Stem-Progenitor Cells) causes myeloid leukemia [7], [12] and MN1 induces proliferation and inhibits myeloid differentiation of both mouse and human HSPC. [13] The differentiation inhibitory and proliferative effects of MN1 can be prevented by re-introduction of CEBPA. [13].

Although the transforming capacity of MN1 is well established, the molecular mechanisms and pathways that regulate its leukemogenic activity remain elusive. We hypothesized that identification of the domains within MN1 contributing to its leukemic activity, and determination of the gene expression profiles of cells that ectopically express MN1 or a mutant lacking leukemic activity, could provide more in-depth information about the genetic programs involved in MN1-induced myeloid leukemia. Here, we mapped the regions of MN1 that confer myeloproliferative, differentiation-inhibitory and leukemogenic activity on mouse HSPC. In addition, we could distinguish expression profiles associated with MN1’s myeloproliferative and differentiation inhibitory effects from its myelo-transforming activity. This was accomplished by comparing the transcriptome of highly purified common myeloid progenitors (CMP) overexpressing MN1 or a MN1 deletion mutant, which induced myeloproliferation and prevented myeloid differentiation *in* *vitro,* but did not cause leukemia in mice.

## Materials and Methods

### Ethics Statement

This study was carried out in accordance with the recommendations in the Guide for Care and Use of Laboratory Animals of the National Institutes of Health. The protocol was approved by the Institutional Animal Care and Users Committee of St Jude Children’s Research Hospital (Approved protocol Number: 209-100171-04/12). All efforts were made to minimize suffering.

### Plasmids and Viral Packaging

Full-length human *MN1* cDNA [14] was cloned into MSCV-IRES-GFP (MIG) retroviral vectors as an EcoRI fragment. To ensure nuclear localization, we added a C-terminal SV40-NLS (PKKKRKVG) to all MN1 mutants used in this study. Deletion mutants of MN1 (Δ1260–1320, Δ12–228, Δ18–458, Δ570–950, Δ570–1010, Δ570–1080, Δ570–1109, Δ570–1119, Δ570–1175, Δ570–1209, Δ570–1273, Δ458–560+Δ570–1119, Δ397–Δ560+570–Δ1119, and Δ50–189+Δ570–1119) were generated using suitable restriction enzyme sites and deleted restriction enzyme fragments were replaced by short double stranded synthetic oligonucleotides to maintain the MN1 open reading frame across the deletion.

Using these constructs we generated VSVg-pseudotyped retrovirus as described. [13].

### Generation of MN1 and MN1-mutant Cell Lines

The U937 cell line [15] was maintained and transduced with the corresponding MIG viruses for two days as described (2.5×10^5^/well, 12 well plate). [13] GFP^+^ cells were sorted using FACS and differentiation was induced with vitamin-D_3_ (100 nM; D1530, Sigma-Aldrich, St. Louis, MO, USA), all-trans retinoic acid (ATRA, 1 uM) (R2625, Sigma-Aldrich) or the same amount of vehicle (100% Ethanol) for 3 days. Differentiation was assessed using FACS analysis of CD11b expression.

### FACS Analysis

FACS analysis or collection of GFP^+^ cells was performed as described. [13] Anti-human CD11b-APC and CD14-PE were purchased from Miltenyi Biotec (Auburn, CA, USA), anti-mouse antibodies (Mac1-PE, Gr1-APC, Sca1-PerCP-Cy5-5) from BD Biosciences (Franklin Lakes, NJ, USA), and ckit-APC-Alexa750 from eBiosciences (San Diego, CA, USA). For isolation and analysis of CMP, megakaryocyte erythroid progenitors (MEP) and granulocyte macrophage progenitors (GMP), mouse bone marrow cells were washed, suspended in blocking solution [0.2 mg/ml human gamma globulin in phosphate-buffered saline (PBS)] for 20 minutes on ice, and washed and stained with PE-conjugated CD34 antibody (BD Biosciences) for 30 minutes on ice. After washing, cells were stained with an antibody mixture consisting of PerCP-Cy5.5-conjugated Sca-1 antibody (eBioscience), PE-Cy7-conjugated CD127 (IL7R) antibody (eBioscience), Alexa700-conjugated FcgammaR antibody (BD Biosciences), APC-Alexa780-conjugated c-kit antibody (eBiosciences), and a mixture of PE-Cy7-conjugated lineage-specific antibodies (CD4, CD8, B220, Gr1, and TER119, all from BD Biosciences) for 30 minutes on ice. After washing, cells were suspended in PBS/10% FBS and analyzed and sorted using a FACS Aria II cell sorter (BD Biosciences). Wild type freshly isolated mouse bone marrow cells were used to set-up the CMP, GMP and MEP gates. [16].

### Western Blot and Immunocytochemistry

Total cell lysates were prepared in 2% SDS containing protease inhibitors (Sigma) followed by three 15-second pulses of sonication. Lysates (from 4×10^5^ cells) were separated on 4–20% stain free Tris-HCL polyacrylamide gel (PAGE), (Biorad, Hercules, CA, USA) and transferred to polyvinylidene diflouride membranes (Millipore, Bedford, MA, USA). Membranes were incubated with anti-human MN1 antibodies recognizing either the N-terminus [7] or C-terminus of the protein (sc-27349; Santa Cruz, Santa Cruz, CA, USA) followed by incubation with horseradish peroxidase (HRP)-conjugated secondary antibodies. Protein bands were visualized with chemiluminescence (G&E Healthcare Biosciences Pittsburgh, PA, USA). A stain free gel imaging system (Biorad) or Anti-human ACTIN antibody (4968; Cell Signaling, Danvers, MA, USA) were used as a control for equal gel loading.

Immunocytochemistry of the cells using anti-MN1 antibody was performed as described. [7].

### Mouse Bone Marrow Culture

C57Bl6 mice (8 to 11 weeks old) were injected intraperitoneally with 5-fluorouracil (5-FU; 150 µg/gr) (Sigma, F6627). Six days later, BM cells were isolated, washed, and stained with a cocktail of phycoerythrin(PE)-labeled lineage-specific antibodies (CD4, CD8, Mac1, Gr1, Ter119, B220 and NK1.1; BD Biosciences), and the labeled lineage positive (lin^+^) cells were depleted using an AutoMACS magnetic separator (Miltenyi Biotec) according to manufacturer’s specifications. Following 1-day expansion of 5×10^5^ cells/ml in 4 ml per well in a six-well plate at 37°C 5% CO_2_ in growth medium [Iscove’s Modified Dulbecco Medium, 10% heat inactivate d fetal bovine serum (FBS; Fisher Scientific, Pittsburgh, PA, USA), 2 mM glutamine, 100 U/mL penicillin/streptomycin (Invitrogen, Grand Island, NY, USA), 50 ng/ml SCF, 10 ng/ml IL-6 and 10 ng/ml IL-3 (PeproTech, Rocky Hill, NJ United States)], cells were transduced using retronectin-coated (Fisher Scientific) plates with viral supernatants 1∶2 diluted in growth medium supplemented with cytokines. Cells (5×10^5^/ml; 2 ml per well in 6-well plates) were transduced 3 times at 6–12 hours intervals for two consecutive days.

For differentiation assays, cells were expanded for 96 hours after the last retroviral transduction and subjected to a second lineage depletion using the mouse hematopoietic progenitor enrichment kit (Catalog #19756; Stemcell Technologies, Vancouver, BC, Canada) following the manufacturer’s instructions. Cells were maintained in growth medium and marker analysis was performed at Day-0 and Day-2 after the second lineage depletion using FACS.

In a parallel experiment, cells were transduced and maintained in liquid culture in growth medium and seeded at the same density 24 hours after the last transduction (2×10^6^ cells/well in 4 ml in a 6 well plate). Cells were split (2×10^6^ cells/well in 4 ml) 3 times a week and the growth potential of GFP^+^ cells over that of the GFP^−^ cells in the same culture was measured by performing a periodic GFP analysis using FACS at the indicated time points. Methylcellulose assays with GFP^+^ sorted cells were performed as described. [13].

### Microarray Analysis

Two days after the first transduction (after total 5 days in culture) with the indicated retroviruses, mouse CMP/GFP^+^ cells were collected into TRIzol using FACS and RNA was isolated according to the manufacturers’ protocol. RNA was isolated from bulk bone marrow cells of 2 leukemic MN1-mice using TRIzol and used in microarray experiments. RNA quality was confirmed by analysis on the Agilient 2100 Bioanalyzer. Total RNA (100 ng) was processed in the St Jude microarray core according to the Affymetrix GeneChip eukaryote two-cycle target labeling protocol (http://media.affymetrix.com/support/downloads/manuals/expression_analysis_technical_manual.pdf). Biotin-labeled cRNA (6 µg) was added to a hybridization cocktail and then processed automatically on an HT MG-430 PM array plate using the Affymetrix GeneTitan system. The HT MG-430 PM array contains 45,037 probesets that interrogate more than 39,000 mouse transcripts. Normalized transcript measures were generated from scan intensity files using the RMA algorithm. [17] A two-fold threshold was applied to identify differentially expressed transcripts among cell populations. Probeset annotations were obtained from the Affymetrix NetAffx website (http://www.affymetrix.com/analysis/index.affx). Gene Ontology and pathway enrichment analysis was performed using the DAVID bioinformatics resources [18] (http://david.abcc.ncifcrf.gov/). Gene Set Enrichment Analysis (GSEA) [19] was also performed using canonical pathways obtained from the Broad Institute (C2), Ingenuity Pathways and GeneGo Pathways.

The microarray data of this study have been deposited in GEO under accession no GSE38767.

### Bone Marrow Transplantation and Analysis of Mouse Tissues

Wild type C57Bl6 mice (8–12 weeks old) were used as donors and recipients. Lineage depleted bone marrow cells were isolated, cultured, and transduced as described above. At day 5 of culture, which included the retroviral transductions, cells were harvested, washed once with PBS and diluted in PBS containing 100 U/ml heparin (APP Pharmaceuticals, Schaumburg, IL USA). Lethally irradiated mice (950 rad at 60 rad/min with a cesium irradiator) received transplants 24 hours after irradiation of 5×10^5^ unsorted cells per mouse (tail vein injections). Mice were maintained on Baytril-water for 21 days, starting immediately after irradiation. Complete blood counts were performed with a Hemavet 3700 (Drew Scientific). Peripheral blood samples were analyzed for the presence of GFP using FACS at the indicated times. Tissues were collected, processed, and stained with antibodies against GFP (A11122; Invitrogen) and MPO (A0398; DAKO Carpinteria, CA, USA), and analyzed as described. [20] Cells were also analyzed for expression of CD3, GATA1, and B220 (data not shown).

### Statistical Analysis

Two-way or one-way ANOVA analysis, Kaplan-Meier survival plots and comparison of Kaplan-Meier plots (log-rank (Mantel-Cox) test) were performed using the GraphPad Prism, version 4.0 c for Mac (GraphPad Software; www.graphpad.com).

## Results

### Identification of MN1 Sequences Inhibiting Myeloid Differentiation of U937 Cells

**Figure 1:**
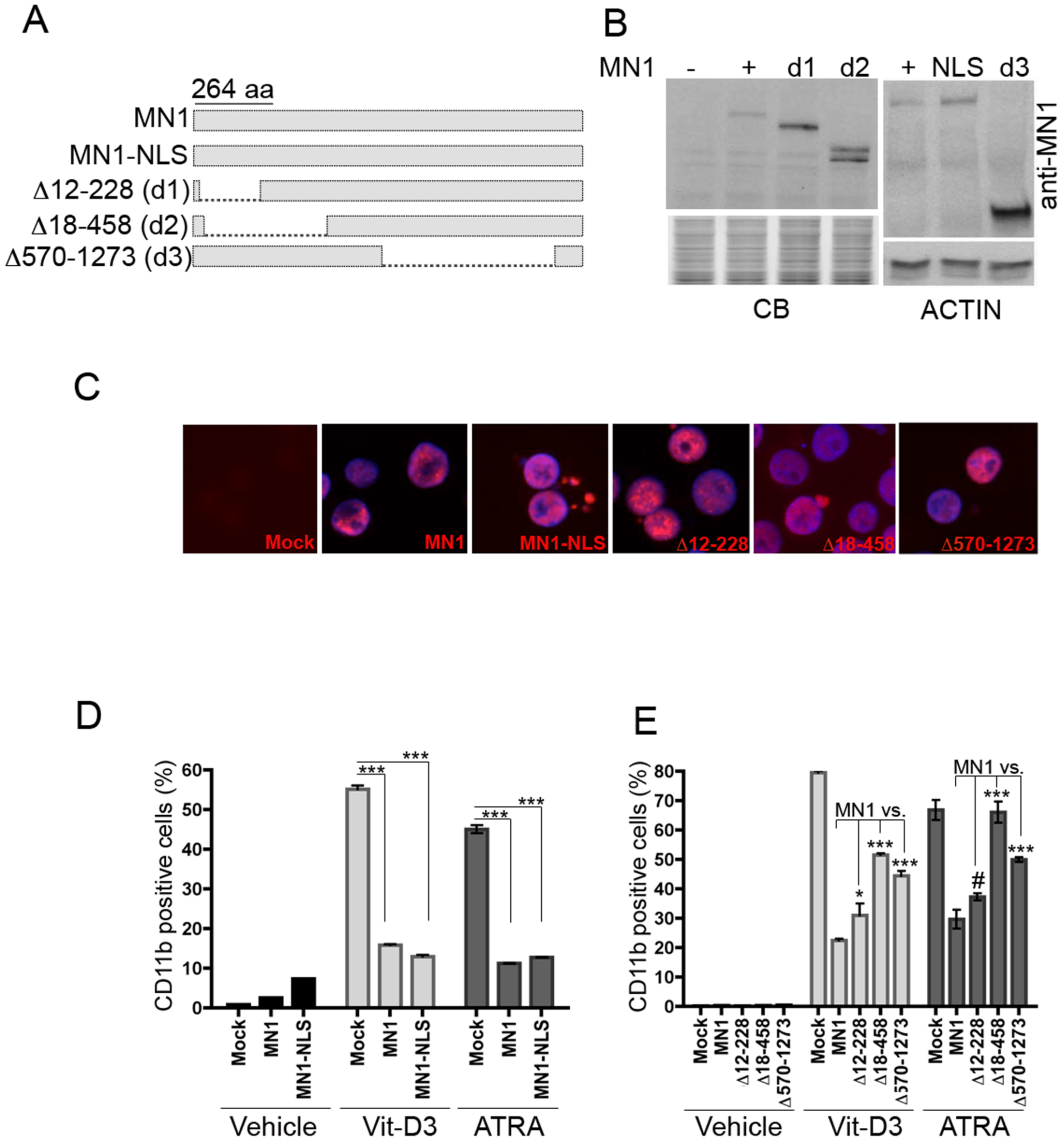
Effects of MN1-deletion mutants on myeloid differentiation of U937 cells. (A) Schematic representation of MN1 and MN1-deletion mutant proteins. (B) Western blot analysis of GFP-sorted U937 stable cell lines overexpressing the indicated proteins. Figure shows immunoblotting results of anti-MN1 antibodies recognizing either the N-terminus (top left panel) or C-terminus of the protein (top right panel) and anti-human ACTIN antibody (bottom right panel). The commassie-blue stained image of the PAGE gel (bottom left panel; CB) and a pan-actin blot (bottom right panel; ACTIN) are shown as loading controls. (C) Immuno-cytochemical analysis of the above cells using the same MN1-antibodies (red). Blue shows nuclear staining with 4,6-diamidino-2-phenylindole (DAPI). Images were captured with an Olympus BX-50 microscope (equipped with UPlanFL 40×/0.75 numerical apertures with a SPOT camera and SPOT Advanced Imaging software of Diagnostic Instruments. The original magnification was x400. (D, E) FACS analysis showing CD11b expression of indicated U937 stable cell lines 3 days after treatment with vehicle, vitamin D_3_ (Vit-D3) or ATRA (mean ± SEM of duplicates; *P<.05, ***P<.001, ^#^not significant).

*MN1* causes acute myeloid leukemia when overexpressed in mouse HSPC. [7], [12] Its overexpression also caused impaired myeloid differentiation, increased proliferation, and increased self-renewal of primary mouse or human CD34^+^-HSPC *in vitro*. [13] Based on data of MN1 sequences essential for the transforming activity of the MN1-TEL fusion protein [21], we first tested the oncogenic activity of MN1 deletion mutants Δ12–228, Δ18–458 and Δ570–1273 (Figure 1A). To ensure that mutants would localize to the nucleus [14], we added a SV40-NLS to all MN1 constructs. Addition of the NLS did not affect MN1 function as overexpression of full length MN1 or MN1-NLS similarly impaired vitamin-D_3_ or ATRA induced monocytic or granulocytic differentiation, as judged by expression of the differentiation marker CD11b (Figure 1D). We confirmed the ectopic expression and nuclear localization of each MN1 mutant in FACS-sorted GFP^+^-U937 cell lines using western blot (Figure 1B) and immunocytochemistry (IC) (Figure 1C). The three deletion mutants showed a differentiation inhibitory effect with the Δ12–228 mutant showing the strongest inhibition (1.2 and 1.3-fold better differentiation than MN1-cells, in response to vitamin D_3_ and ATRA, respectively) and the Δ18–458 mutant the least inhibition (2.2 fold improvement in vitamin D_3_ response and complete recovery of the ATRA response)(Figure 1E). This indicated that aa residues between 12–228 contributed to but were not essential for the differentiation block observed in U937 cells.

### MN1 Increases the Frequency of CMP *in vitro*

**Figure 2:**
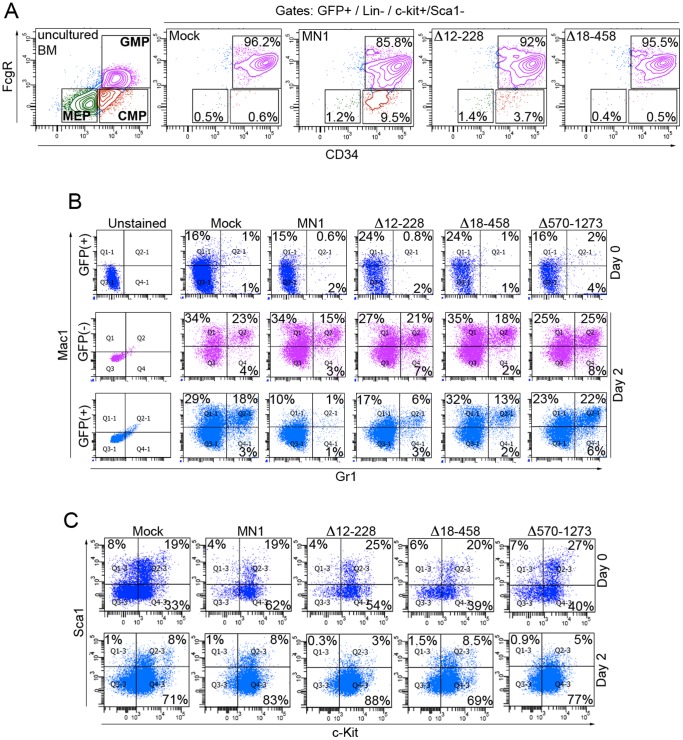
Deletion of aa residues between 18–458 or 570–1273 of MN1 restores MN1-impaired myeloid differentiation of mouse HSPC *in vitro.* (A) FACS analysis showing the distribution of MEP, CMP and GMP populations in lineage depleted mouse bone marrow HSPC that were transduced with retroviruses expressing GFP alone (mock), MN1, or the indicated MN1-deletion mutants. Freshly isolated bone marrow cells were used to set up the gates. Figure shows one representative results from 3 independent experiments. (B) Mouse HSPCs transduced with indicated retroviruses were subjected to a second lineage depletion 96 hrs after transduction (Day-0) and cells were cultured for an additional 2 days (Day-2). FACS analysis shows Mac1 and Gr1 expression of unsorted cells at Day-0 and Day-2 of culture. (C) FACS analysis showing the Sca1 and c-Kit expression of the GFP (+) cells in panel B.

Mouse HSPC transduced with retroviruses encoding full length or the three MN1 deletion mutants were analyzed for their *in vitro* differentiation potential in response to cytokines. FACS analysis performed 2 days after the first transduction showed that, relative to mock transduced cells, the percentage of CMP cells in the GFP^+^/Lin^−^/c-Kit^+^/Sca1^−^ fraction in both MN1 and Δ12–228-expressing cells was increased, albeit more modestly in the latter (Figure 2A). This increase slightly enlarged the downstream MEP population, but decreased the respective downstream GMP populations by 10.4% and 4.2%, respectively (Figure 2A). In contrast, cells expressing the Δ18–458 mutant showed the same CMP, MEP and GMP distribution as mock transduced cells (Figure 2A).

To test the differentiation potential of transduced cells beyond the GMP stage, we repeated the same experiment and included the Δ570–1273 mutant. Following transduction and a 96 hours expansion of the cells, a second lineage depletion was performed (referred to as Day-0) to purge cells that had differentiated during the transduction and expansion process. FACS analysis of Day-0 cells showed similar low levels of Mac-1 and Gr-1 expression in all samples, indicating efficient depletion of maturing myeloid cells from each culture (Figure 2B). Differentiation of the cells was evaluated 48 hours later (Day-2). FACS results of Day-2 MN1-cells or MN1-mutant-cells compared with mock-cells did not show any increase in the Sca-1^+^/c-Kit^+^ population, which is enriched in hematopoietic stem cells (Figure 2C). As expected, 50% of mock-transduced cells expressed myeloid differentiation markers (Mac-1, Gr-1 or Mac-1/Gr-1) at Day-2, which was 2.7 fold higher than at Day-0 (Figure 2B). In contrast, only 12% of MN1-transduced cells and 26% of Δ12–228-cells expressed these markers at Day-2. The Δ18–458 and Δ570–1273 cells showed fully restored differentiation with 47% and 51% of cells expressing myeloid differentiation markers, respectively (Figure 2B). The GFP-negative population in each culture served as an internal control and expressed equal levels (47%–57%) of myeloid markers in all samples at Day-2 (Figure 2B). Collectively, Δ12–228 cells displayed a less pronounced inhibition of myeloid differentiation than MN1 cells, while Δ18–458 or Δ570–1273 cells showed no inhibition of differentiation in this assay.

### Mapping of Sequences Essential for MN1-mediated Myeloproliferation and Self-renewal *in vitro*

**Figure 3:**
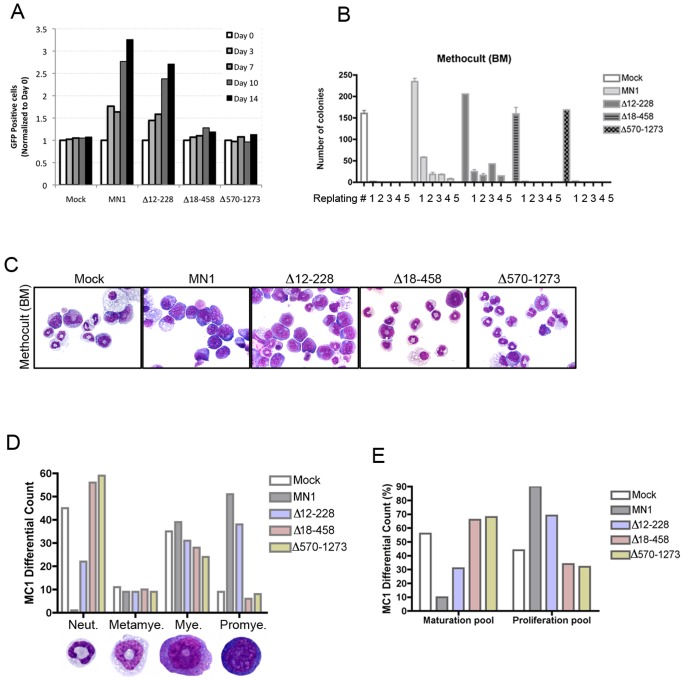
Amino acid residues 12–228 of MN1 are not required for its proliferative and self-renewing activity *in vitro*. (A) Periodic GFP analysis was performed at indicated times of liquid cultures using FACS. GFP percentage at Day-0 of analysis (4 days after the last transduction) in each sample was set to 1 and the results of all indicated time points were calculated relative to Day-0. Graph shows one representative analysis of three independent experiments. (B) Methylcellulose assays showing colony numbers after serial replating (1 to 5) of linage-depleted mouse HSPCs transduced with the indicated retroviruses (mean ± SEM of duplicates). (C) May-Grunwald/Giemsa stained images of cytospins of the cells in panel B (after the first methylcellulose culture). (D, E) Differential-count of cytospins is shown in panel C. A total of 100 cells were counted in each sample. The maturation pool includes neutrophils and metamyelocytes whereas the proliferation pool comprises myelocytes and promyelocytes. The images in panels C and D were captured with an Olympus BX41 microscope, equipped with SPOT Insight Color Mosaic 2 MP camera and SPOT imaging software (original magnification x1000).

Given that sequences in the N-terminal (aa 18–458) and C-terminal (aa 570–1273) regions of MN1 were required to impair the myeloid differentiation of primary mouse HSPC, we tested if these same regions were important for MN1-mediated myeloproliferative activity. Liquid culture of MN1 and Δ12–228-transduced mouse HSPC for 14 days showed increased proliferation, outgrowing the co-cultured uninfected cells as shown by sequential FACS analyses over time. The percentage of GFP^+^ cells increased by 3.2-fold in the MN1-transduced cultures (from 28% at Day-0 to 91% at Day-14), by 2.7-fold in Δ12–228-transduced cultures (35.8% at Day-0 and 97.2% at Day-14) but did not increase in the Δ18–458, Δ570–1273, or mock-transduced cultures (Figure 3A). Previously we showed that forced MN1 expression increases proliferation of mouse [7] and human HSPC *in vitro*. [13] Our current results suggest that sequences within aa 18–458 and aa 570–1273 are essential for this MN1-induced effect.

In addition to abnormal growth and impaired differentiation, acquisition of increased self-renewal capacity by HSPC is essential for the development of leukemia. Therefore, we evaluated the *in vitro* self-renewal potential of the MN1-deletion mutants using a methylcellulose (MC) serial-replating assay. Consistent with the *in vitro* proliferation assays, Δ12–228-cells recapitulated the effect of full length MN1 and showed increased replating and proliferation capacity, as was judged by both the increased number and size of the colonies, whereas the replating activity of the cells transduced with vector, Δ18–458 or Δ570–1273 mutants was exhausted after the initial MC culture (Figure 3B). Giemsa staining and differential counts of cytospin preparations from the MC-1 plates demonstrated a 5.6-fold and 4.2-fold increase in the number of promyelocytes in MN1 and Δ12–228-cultures, respectively (Figure 3C and 3D). Similar to the liquid culture results (Figure 2B), MN1 cells did not mature into neutrophils (1% in MN1 vs. 45% in mock; Figure 3D). The Δ12–228 mutant partially reproduced this effect, which, compared with mock-cells, showed a 2-fold inhibition of maturation into neutrophils (Figure 3D). In contrast, Δ18–458 and Δ570–1273 cells showed a moderate increase in the number of mature neutrophils compared to mock-transduced cells (Figure 3D). Therefore, both MN1 and Δ12–228 increased the pool of proliferating promyelocytes and myelocytes at the expense of the pool of mature metamyelocytes and neutrophils (Figure 3E). In conclusion, deletion of aa 12–228 of MN1 moderately decreased its ability to block myeloid differentiation in methylcellulose assays whereas the self-renewal (replating capacity of colonies) and proliferative activity of MN1 were maintained. Thus, sequences comprised within aa 18–458 and aa 570–1273 of MN1 are necessary to induce aberrant self-renewal, increased proliferation, and impaired differentiation of primary mouse HSPC *in vitro*.

### Sequences in both the N-terminal and C-terminal Regions of MN1 are Required for its Leukemogenic Activity

**Figure 4:**
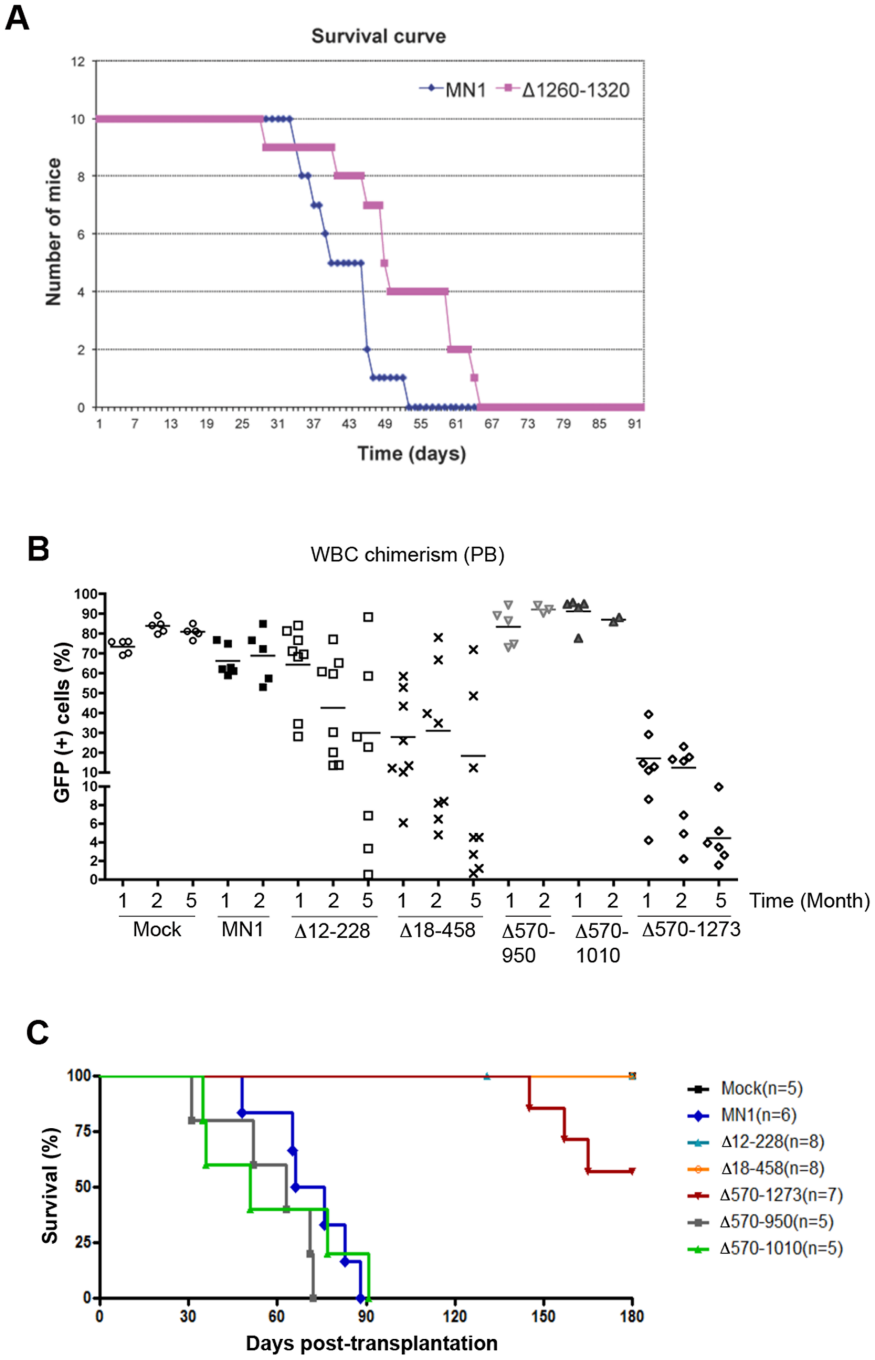
Deletions in MN1 that interfere with its leukemogenic activity. (A) Comparison of *in vivo* leukemogenic activity of MN1 and Δ1260–1320 transduced bone marrow after transplantation in 10 lethally irradiated mice each. (B) FACS GFP analysis of the peripheral blood (PB) of mice that received transplants of BM expressing MN1 or the indicated MN1 mutants at the indicated time points after transplantation. WBC: White blood cells. (C) Kaplan-Meier survival plots of mice transplanted with bone marrow cells transduced with each indicated retrovirus.

The MN1-TEL1 fusion protein only contains MN1 sequences encoded by its first exon but not the 60 aa encoded by its second exon [14]. Therefore we tested the oncogenic activity of the first exon of MN1 (Δ1260–1319) versus that of full-length MN1 in our *in vivo* leukemogenesis assay. Mice receiving transplants with MN1 or Δ1260–1319 transduced bone marrow both developed AML with or without maturation between 35–52 and 30–62 days post transplant, respectively (Figure 4A). Next, we tested the *in vivo* transforming capacity of the Δ12–228, Δ18–458 and Δ570–1273 MN1-mutants. Periodic FACS analysis of the GFP content of peripheral blood samples showed successful engraftment in each group (Figure 4B and Table 1). Mice receiving transplants of MN1-transduced bone marrow developed myeloid leukemia within 48–88 days (median survival 71 days) (P<0.0001, log-rank test; Figure 4C). However, mice receiving mock and Δ18–458 transduced bone marrow remained disease free till the end of the observation period (6 months), while 3 of 7 mice receiving Δ570–1273 bone marrow developed GFP^−^ T cell lymphoma (Figure 4C, see below). Despite its increased self-renewal and myeloproliferative activity *in vitro*, mice receiving transplants of Δ12–228-expressing bone marrow (transduction rate of the transplanted cells were similar to that of full length MN1, Table 1) did not develop leukemia in two independent experiments (Figure 4C). One of 8 mice in Δ12–228-group was found dead at 131 days after transplantation but the cause of death could not be determined due to autolysis of the tissues, and was therefore omitted from the study.

**Table 1:** Percentage of GFP (+) cells at time of transplantation.

	Mice (n)	GFP (%)
**Vector**	5	72
**MN1**	6	14
**Δ12**–**228**	4	18
	4	22
**Δ18**–**458**	2	41
	6	22
**Δ570**–**1273**	7	39
**Δ570**–**950**	5	53
**Δ570**–**1010**	5	52
**MN1** *	10	50
**Δ1260**–**1320** *	10	51
**Other MN1 mutants** #	4–12	10–20

* Experiment shown in figure 4A.

# Experiments shown in figure 5B.

**Figure 5:**
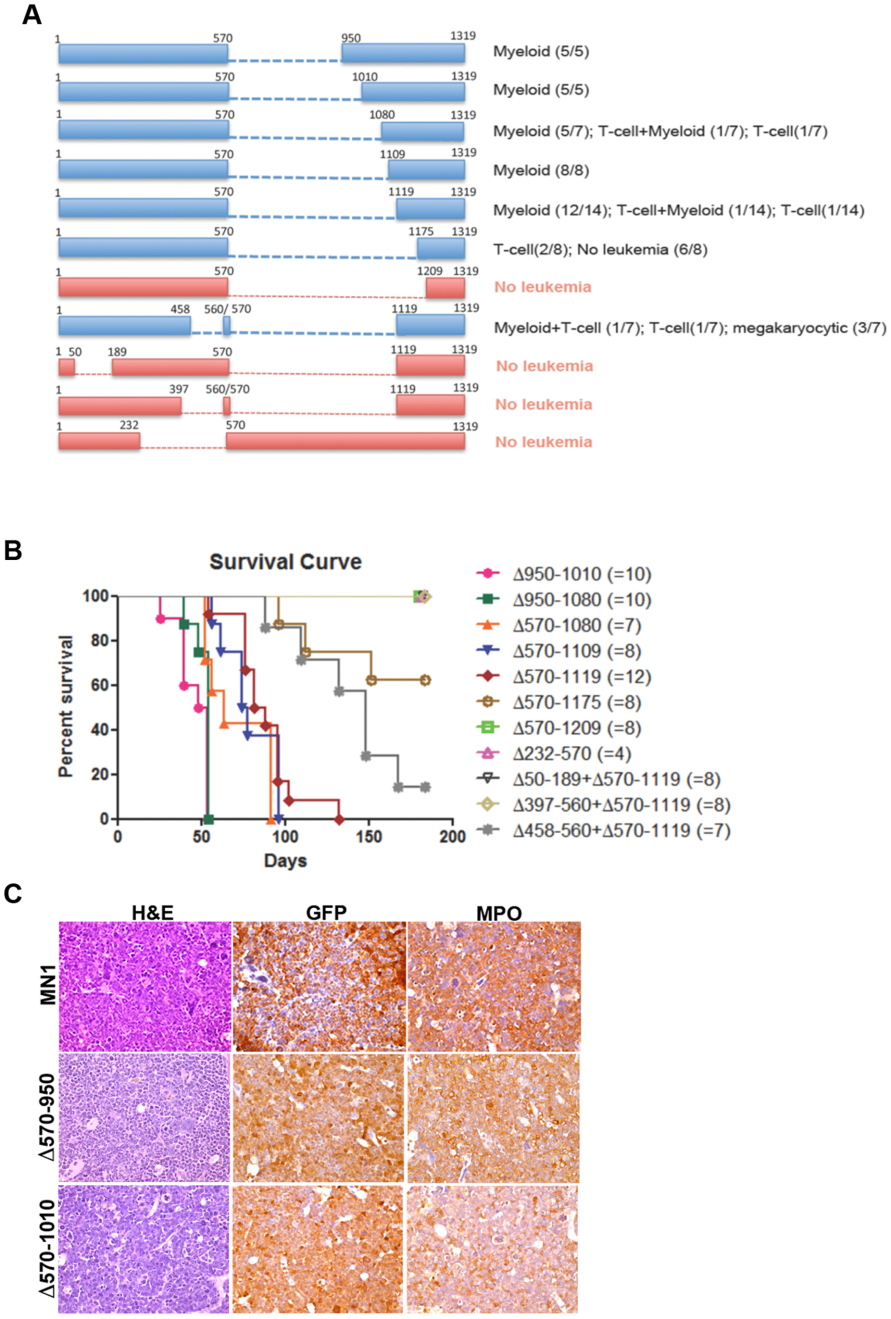
Kaplan-Meier survival curves of mice receiving MN1 or mutant MN1-transduced bone marrow. (A) Schematic representations of MN1 deletion mutants. Mutants depicted in blue do cause leukemia, while mutants in red do not. Leukemia types generated by each mutant are indicated in the Figure. Stippled lines indicate the size of MN1 deletions. (B) Comparison of Kaplan-Meier survival curves of mice receiving transplants of bone marrow transduced with the indicated MN1 deletion mutants. (C) Histological analysis of a bone marrow sample of a representative leukemic mouse in each indicated group. Images were taken at 400X using an Olympus BX41 microscope, equipped with an Insight 2 Mega-pixel Color Mosaic camera, employing SPOT advanced imaging software. H&E = hematoxilin & eosin staining, GFP = green fluorescent protein staining, MPO = myeloperoxidase staining.

To further delineate the domains in MN1 essential for leukemogenesis we generated additional deletions in the 18–570 and 570–1273 regions. Given the results with the Δ12–228 mutant we resided to testing the leukemogenic potential of the additional mutants *in vivo* only. To further dissect the 570–1273 region, we generated the Δ570–950 (Figures 5A, 5B), Δ570–1010 (Figures 5A, 5B), Δ570–1080, Δ570–1109, Δ570–1119, Δ570–1175 and Δ570–1209 deletion mutants (Figure 5A) and tested their leukemogenic activity in mice receiving transplants of bone marrow transduced with each mutant. With the exception of the latter two, all mutants caused myeloid leukemia between 30 and 130 days post transplant. Increasing the size of the deletion did not affect the type of AML, which in all cases was with or without maturation. Seven mice expressing mutant MN1 developed T cell lymphoma (one Δ578, one Δ570–1119, two Δ570–1175 and three Δ570–1273 mice) while one (Δ570–1080) developed both T-cell lymphoma and myeloid leukemia. Although the Δ570–1175 and Δ570–1273 mutants developed T cell lymphoma only, it is currently unclear if there is a direct correlation with mutant MN1 expression because four lymphomas were GFP^+^ (one Δ578, one Δ570–1119, one Δ570–1175 and the one Δ578 mixed leukemia/lymphoma) while the others were GFP^−^ (data not shown). Immunohistochemical analysis of selected leukemic bone marrow samples marking the transplanted cells (GFP staining) and myeloid cells (Myeloperoxidase (MPO)) are shown in the Figure 5C.

Next, we delineated the 18–570 region using the Δ232–570, Δ458–560+Δ570–1119, Δ397–560+Δ570–1119, and Δ50–189+Δ570–1119 mutants (Figure 5B). Compared with the Δ570–1119 mutant, the Δ458–560+Δ570–1119 mutant slightly delayed the onset of leukemia and produced combined myeloid leukemia/T-cell lymphoma (1/7), T-cell lymphoma (1/7) and megakaryocytic leukemia (3/7), which were all GFP^+^, while one mouse was found dead and could not be analyzed due to autolysis. The Δ232–570, Δ397–560+Δ570–1119, and Δ50–189+Δ570–1119 mutants failed to develop leukemia in the 6 months observation period (Figure 5B). Together these data indicated that aa 18–458 and 1119–1273 are minimally required for MN1’s myeloid leukemogenic activity.

### Expression Profiling of Purified CMP Populations

**Figure 6:**
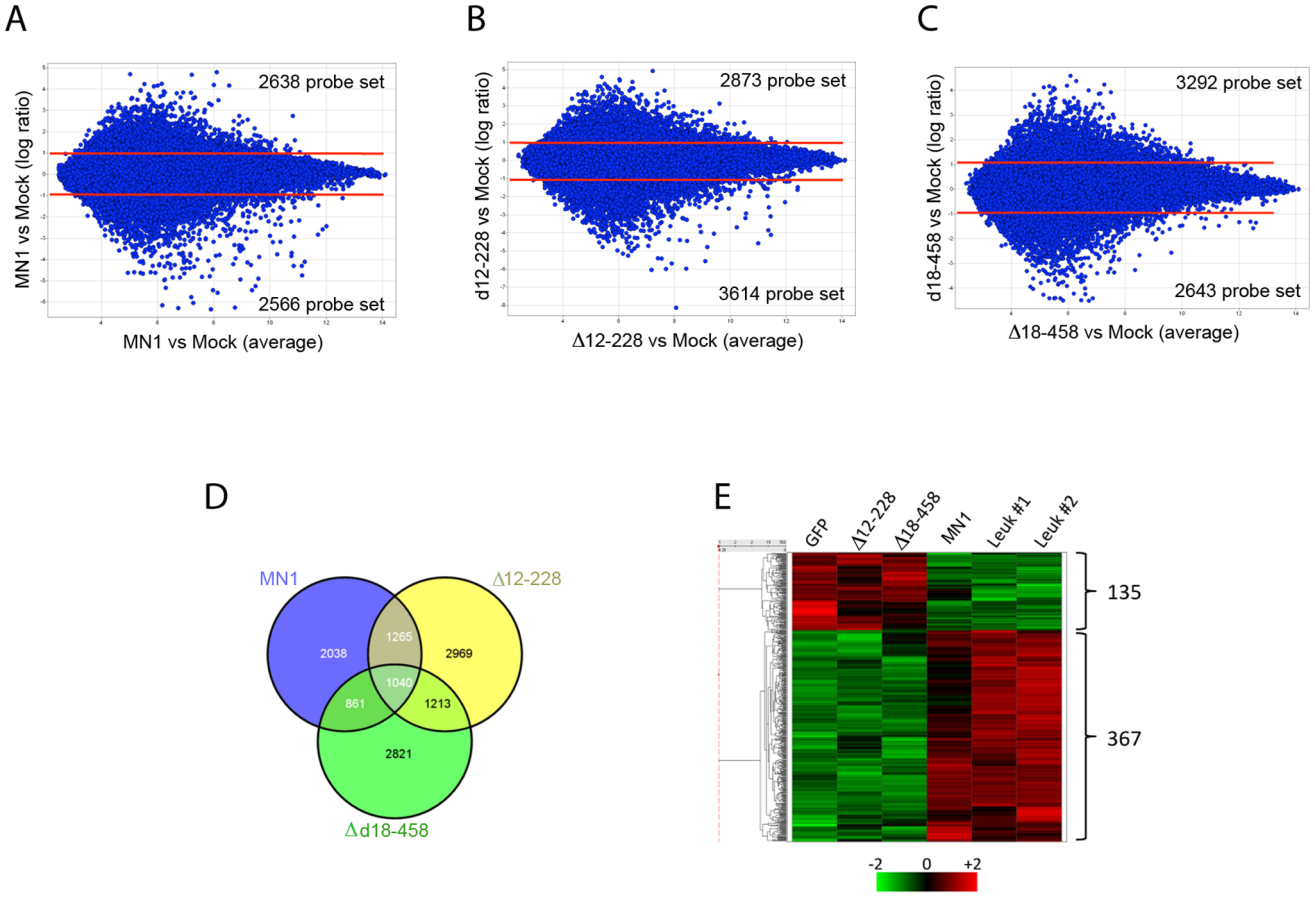
Expression profile analysis of MN1, 12–228 and 18–458 cells. (A, B, C) Pair wise comparison of CMP/GFP^+^ cells transduced with MN1, Δ12–228, Δ18–458 retrovirus with CMP/GFP^+^ cells transduced with empty vector (mock). (D) Venn diagram showing the number of probe sets that are differentially expressed in the pair wise comparison in A, B and C. (E) Hierarchical clustering of 502 transcripts with >2-fold differential expression in MN1 CMP/GFP^+^ cells and two bulk leukemic MN1-bone marrow (Leuk #1, #2) as compared with CMP/GFP^+^ cells transduced with empty vector (GFP). Heat map indicates expression relative to the mean in standard deviation units.

We next sought to distinguish the genetic program mediating MN1’s role in increased self-renewal/proliferation and inhibition of differentiation in vitro from that mediating MN1’s transforming activity. Given that ectopic expression of MN1 increased the frequency of CMP *in vitro*, we performed a microarray analysis using FACS-sorted CMP^+^/GFP^+^ cells expressing vector, MN1, the Δ12–228, or the Δ18–458-deletion mutant. We used DAVID bioinformatics database (http://david.abcc.ncifcrf.gov/) to functionally categorize differentially expressed genes. Pair-wise comparisons for each of the three samples using vector-CMP cells as baseline (Figure 6A, 6B and 6C) with a threshold of >2-fold difference was used to identify differentially expressed transcripts. As shown in the Venn diagram in Figure 6D, 2038 transcripts were differentially expressed in MN1-CMP only and 1265 transcripts were differentially expressed in both MN1 and Δ12–228-CMP, while 1110 of these were regulated in the same direction. Therefore, these 2038 MN1-specific transcripts are potentially involved in MN1-induced myeloid leukemia, whereas the 1110 overlapping transcripts between MN1-cells and Δ12–228-cells may play a role in MN1-induced enhanced myeloproliferation and impaired differentiation of murine bone marrow cells. To identify similarities between the immediate MN1-induced expression profiles and those in leukemic MN1-mice, we determined the differentially expressed transcripts in two bulk leukemic MN1-bone marrow samples using the mock-CMP profile as baseline. Further comparison of CMP-MN1-specific transcripts with the leukemic bone marrow transcripts showed that 502 of 2038 MN1-specific transcripts were differentially regulated in the same direction in the leukemic cells (Figure 6E). Table 2 lists selected transcripts representing potentially important genes. We have also used GSEA to analyze our microarray data. Using canonical pathways (C2 from Broad, Ingenuity, and GeneGo) we did not obtain significant differences (FDR<0.05) between cells transduced with MN1 or the MN1 deletion mutants. Comparison of MN1 vs. GFP comparison indicated that only the BMP2 pathway was significantly activated in MN1 cells (Figure S1). The microarray data of this study have been deposited in GEO under accession no GSE38767.

**Table 2:** Selected genes that are differentially expressed in microarray analysis (fold change).

probe set ID	Gene	CMPMN1 vs Mock	CMPΔ12–228 vs Mock	CMPΔ18–458 vs Mock
1420565_PM_at	Hoxa1	**4.41**	1.89	1.04
1455626_PM_at	Hoxa9	**2.58**	−1.01	−1.03
1421537_PM_at	Hoxd3	**2.11**	1.60	−1.01
1424704_PM_at	Runx2*	**4.23**	1.13	1.04
1443260_PM_at	Meis1#	**2.06**	1.51	1.33
1422734_PM_a_at	Myb*	**2.51**	1.65	1.47
1457193_PM_at	Mll3*	**2.62**	−1.23	−1.17
1452377_PM_at	Mll1*	**6.87**	**5.35**	1.09
1449232_PM_at	Gata1#	−**2.85**	−1.43	1.00
1418982_PM_at	Cebpa	−**2.35**	−**2.87**	1.08
1418901_PM_at	Cebpb	−**3.12**	−**3.36**	−1.48
1423233_PM_at	Cebpd	−**24.25**	−**7.41**	−1.92
1419872_PM_at	Csf1r (M-CSFr)	−**9.99**	−**21.11**	1.01
1419873_PM_s_at	Csf1r	−**7.26**	−**6.36**	−1.04
1420704_PM_at	Csf2ra (GM-CSFr)	−**2.46**	−**2.45**	1.57

# *Regulated in the same direction in CMP-MN1 and in 1 out of 2 (^#^) or 2 out of 2(*) bulk MN1-leukemic BM.

## Discussion

In this study we mapped the minimal regions of MN1 necessary for its leukemogenic activity as well as the regions of MN1 that distinguish its leukemic in vivo activity from its *in vitro* myeloproliferative/self-renewal and differentiation inhibitory activities. Furthermore, we identified early changes in the expression profiles of MN1-CMP cells that presumably contribute to a transformed stage, by performing microarray analysis of highly enriched FACS-sorted-CMP cells. We found that 24.6% of differentially expressed genes in MN1-CMP cells were also regulated in the same direction in leukemic bone marrow cells of MN1-mice.

Our study indicates that integrity of two regions of MN1, comprised within residues 18–458 and 1119–1273, are required for its myeloid leukemic activity, and presence of only one of these regions in the absence of the other is insufficient for *in vivo* transformation. Via which mechanism these regions contribute to the transforming activity of MN1 will be a question that we aim to answer in future studies. We favor the explanation that the deletions remove domains of MN1, which interact with partner proteins that are necessary for its leukemogenic activity, but we cannot exclude other possibilities such as the deletions altering the conformation of the protein thereby inactivating it. As mentioned above, it is currently unclear if some of the deletion mutants of MN1 directly generate T-cell lymphoma, given that 4 out of 8 cases were GFP^+^ and the remaining 4 cases GFP^−^. Because we were interested in MN1’s function as a myeloid disease gene, we did not investigate this issue further and will address this observation in a future study. It is intriguing that the Δ458–560+Δ570–1119 mutant was unique in that it also caused GFP^+^ megakaryocytic leukemia, indicating a correlation between the presence of aa 458–560 and a switch from myeloid to megakaryocytic leukemia.

We hypothesized that deletion of residues 12–228 of MN1 would affect the transcriptional activation of targets that modulate MN1-induced leukemogenesis, given that this mutant does not exhibit *in vivo* leukemic activity. We reasoned that comparison of the transcriptomes of cells expressing full length MN1 or a non-leukemogenic MN1-mutant could provide a more refined insight into the genetic program causing MN1-induced myeloid leukemia. Given that MN1 overexpression expanded the CMP population *in vitro* (Figure 2A), and transforms CMP but not GMP cells *in vivo* [22], we used microarray analysis of highly purified CMP cells overexpressing MN1, Δ12–228 or Δ18–458 to identify crucial transcriptional targets of MN1.

Microarray expression profiling identified 2038 transcripts that discriminate MN1-CMP cells from two of the mutants (Δ12–228 or Δ18–458) that do not exhibit *in vivo* transforming activity. Hoxa9, Meis1, Mll, Runx2, and Myb were among these differentially upregulated genes (Table 2). All these genes play a well-documented role in the transformation of myeloid cells and are frequently involved in myeloid leukemia.[23]–[26] Interestingly, the activity of Meis and Hox genes renders mouse CMP cells susceptible to MN1 overexpression [22], while MN1 (ref [7]) or Runx2 (ref [26]) overexpression cooperates with Cbfb-SMMHC-induced mouse AML. Moreover, important regulators of myeloid differentiation including Cebp-α, Cebp-β, and the M-CSF and GM-CSF receptors were down regulated in both MN1-CMP and Δ12–228-CMP populations (Table 2). Disruption of Cebp-α in mice blocks the transition from CMP to GMP, which not only impairs granulopoiesis, but also enhances the self-renewal activity of hematopoietic stem cells. This generates increased numbers of myeloblasts in the bone marrow but does not cause leukemia [27] alone, but does collaborate with other events in the leukemogenic process. [28] Nonetheless, Cebp-α deficient cells can generate granulocytes in response to IL3 and/or GM-CSF in a Cebp-β-dependent manner. [29] Therefore, besides the many genes that are deregulated in mouse MN1-CMP-cells immediately after transduction, it is tempting to speculate that combined altered expression of Hoxa9/Meis1/c-myb and Cebps are at least partially responsible for the MN1-induced leukemic transformation. Insufficient expression of Cebp’s and other myeloid differentiation factors (Table 2) in Δ12–228-cells may impair differentiation and enhance proliferation *in vitro*. However, compared with MN1-cells Δ12–228-cells show reduced Hoxa9/Meis1/c-myb expression, which despite its induced reduction in differentiation factor expression, may render this mutant inadequate to induce leukemia in mice (Figure 4C). The higher expression of Hoxa9/Meis/Myb in MN1-cells most likely also contributes to the more pronounced differentiation defect of these cells (Figures 2 and 3). [25] Downregulation of CEBP-α expression is an important aspect of MN1-imposed changes in CD34^+^ human HSPC, given that forced Cebp-α expression (both U937-MN1 and HSPC-MN1) reversed the MN1-induced defects in myeloid differentiation and proliferation *in vitro*. [13].

**Figure 7:**
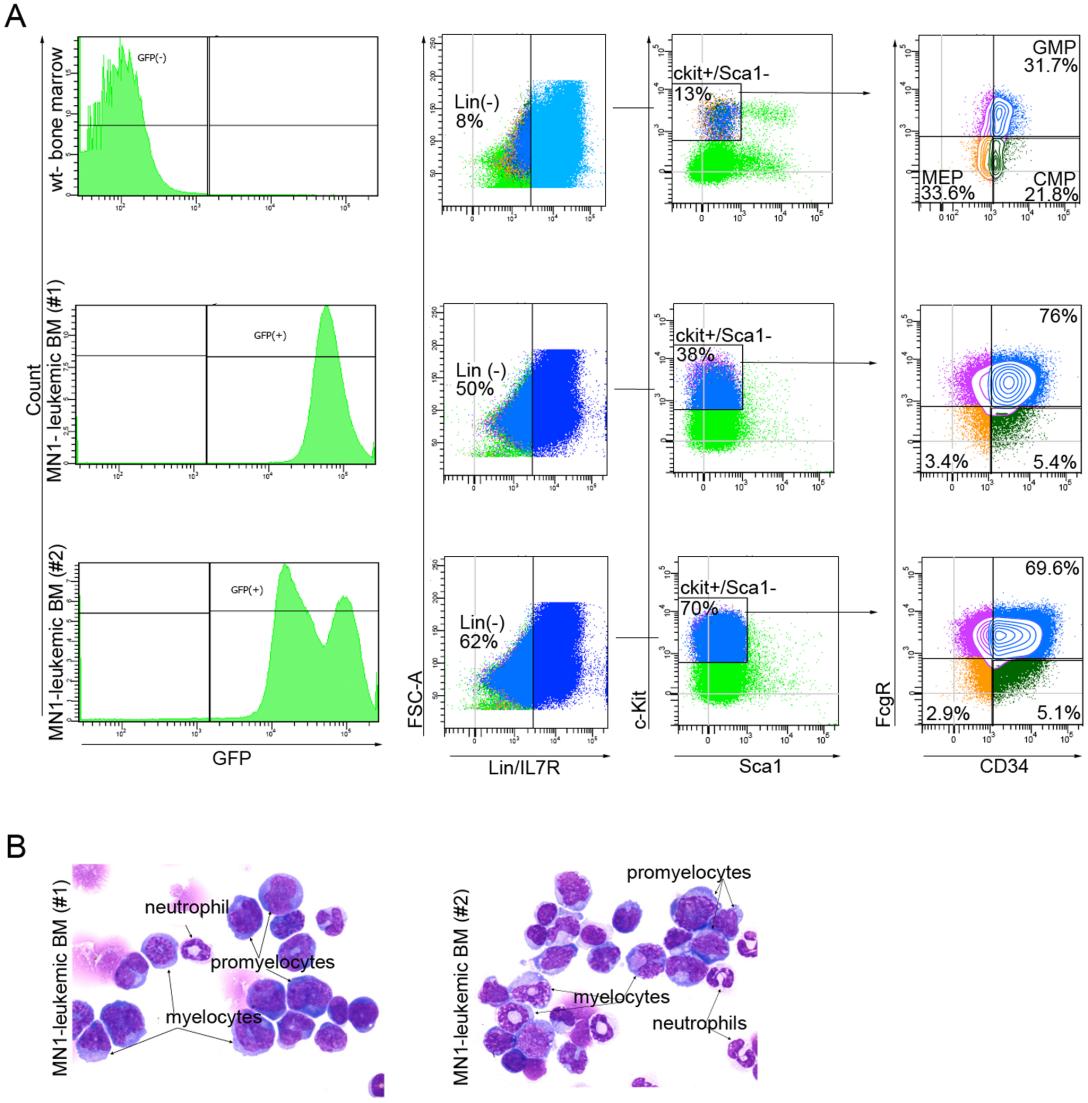
FACS analysis of leukemic bone marrow samples of MN1-mice. (A) FACS analysis of bone marrow samples from a wild type (wt-bone marrow) and two moribund MN1-mice to determine the size of the CMP, MEP and GMP populations. GFP^−^ and GFP^+^ gates were used for wild type or MN1-bone marrow cells, respectively. (B) Giemsa staining of leukemic bone marrow cells of the same mice as in A showing their morphology. Arrows indicate different myeloid cell types.

In our transplantation experiments we used unfractionated and unsorted mouse HSPC that were transduced with MN1-retrovirus. Analysis of the bone marrow samples of leukemic MN1-mice showed a dramatic increase in lin^−^ (8% vs. 50% and 62% in MN1) and c-Kit^+^/Sca1^−^ cells (13% vs. 38% and 70% in MN1) (Figure 7A) when compared to that in wild type mice, which resulted in an increase in absolute numbers of MEP, CMP and GMP cells. Distribution of these three populations in the bone marrow of MN1-mice was skewed towards the GMP (Figure 7A). This suggests that MN1 more severely impairs terminal differentiation downstream of the GMP *in vivo*, giving rise to accumulation of cells that are arrested at that stage, which is in agreement with our *in vitro* findings (Figure 2B).

Future studies that will identify the proteins interacting with MN1, the domains within MN1 required for their interaction, and validation of the role of these potential players in MN1-induced leukemia will further increase our understanding of the precise role of MN1 overexpression in AML. These studies will also form the basis for the design of targeted therapeutic approaches to better treat patients overexpressing MN1 in their leukemic blasts.

## Supporting Information

Figure S1 (TIF)
