# Supplementary figures and images for: Mapping of MN1 Sequences Necessary for Myeloid Transformation
Source: PLoS One. 2013 Apr 23;8(4):e61706. doi: 10.1371/journal.pone.0061706 (PMC3634013; doi:10.1371/journal.pone.0061706)

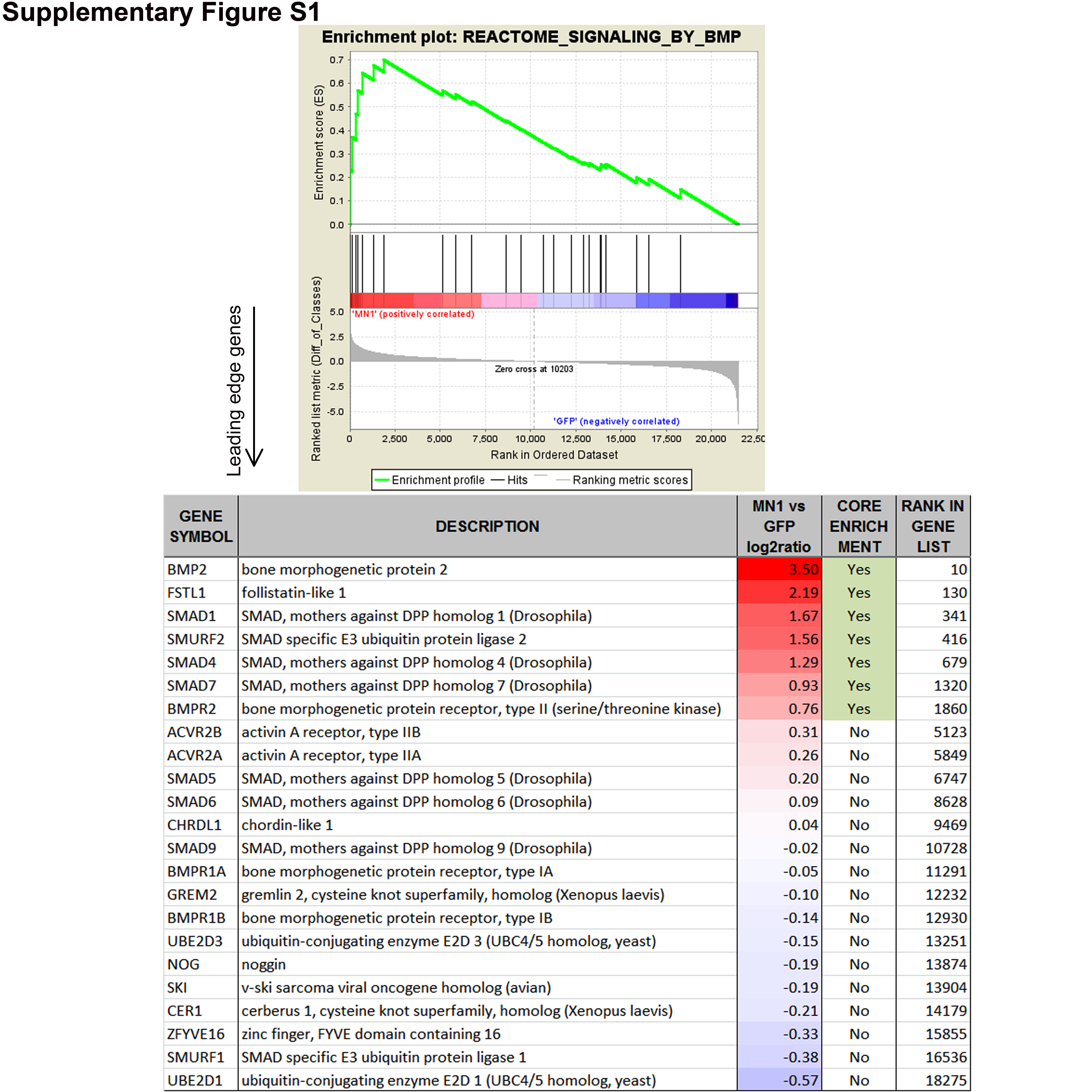

Supplement: Figure S1 — (TIF) [file pone.0061706.s001.tif]
